# Shear Horizontal Surface Waves in a Layered Piezoelectric Nanostructure with Surface Effects

**DOI:** 10.3390/mi13101711

**Published:** 2022-10-11

**Authors:** Lele Zhang, Jing Zhao, Guoquan Nie

**Affiliations:** 1 Hebei Key Laboratory of Mechanics of Intelligent Materials and Structures, Department of Engineering Mechanics, Shijiazhuang Tiedao University, Shijiazhuang 050043, China; 2 Department of Architecture, Shijiazhuang Institute of Railway Technology, Shijiazhuang 050041, China

**Keywords:** shear horizontal surface wave, piezoelectric nanostructure, dispersive behavior, surface effect, size-dependence

## Abstract

This work aims to provide a fundamental understanding on the dispersive behaviors of shear horizontal (SH) surface waves propagating in a layered piezoelectric nanostructure consisting of an elastic substrate and a piezoelectric nanofilm by considering the surface effects. Theoretical derivation based on the surface piezoelectricity model was conducted for this purpose, and analytic expressions of the dispersion equation under the nonclassical mechanical and electrical boundary conditions were obtained. Numerical solutions were given to investigate the influencing mechanism of surface elasticity, surface piezoelectricity, surface dielectricity, as well as the surface density upon the propagation characteristics of SH surface waves, respectively. The results also reveal the size-dependence of dispersive behaviors, which indicates that the surface effects make a difference only when the thickness of the piezoelectric nanofilm stays in a certain range.

## 1. Introduction

Surface acoustic wave (SAW) devices, including sensors, oscillators, filters, photodetectors, etc., are widely employed in various fields for signal processing and sensing due to their high sensitivity and low energy dissipation [1,2,3]. The typical structure for SAW devices is a piezoelectric thin film deposited on a sublayer of another material. When an alternating electric field is applied to the piezoelectric layer, the SAWs can be generated because of the piezoelectric effect and then propagate along the surface either as Rayleigh modes or shear horizontal (SH) modes. To better provide guidance for design and applications of the SAW devices, many efforts have been devoted to investigating the propagation mechanisms of surface waves in layered piezoelectric composite structures. Wu et al. analyzed SH and Rayleigh SAWs in (100) AlN film on (111) diamond substrate, and some excellent acoustic properties were predicted theoretically [4,5]. Liu et al. investigated the dispersive behaviors of SH surface waves propagating in a layered structure consisting of a transversely isotropic piezoelectric film over an isotropic half-space, where the interface is modeled by the shear-lag model to describe the imperfect bonding [6].

In recent years, the rapid development of nanoscience and assembly technology makes it possible to fabricate novel nanoscale SAW devices with fast response and high sensitivity. For example, a ball SAW hydrogen gas sensor employing a sensitive PdNi nanofilm has the widest concentration detection region and short response time (less than 2 s) [7,8]. These advantages mainly attribute to the smaller dimensions combined with a dramatically increased ratio of surface area to volume and strong binding properties of atoms near the surface. Therefore, it is essential to understand the physical and mechanical behaviors of SAW nanodevices with the consideration of surface effects. To date, the methods used for studying the surface effects of nanomaterials and nanostructures can be generally classified into three broad categories: experimental characterization, atomistic simulations, and theoretical analysis. In view of the fact that controlled experiments at nanoscale are extremely difficult, and atomistic simulations are limited by computation capabilities, many researchers have resorted to theoretical modeling through modifying the conventional continuum models. The most famous one is the so-called surface elasticity model established by Gurtin and Murdoch, in which a surface is regarded as a two-dimensional continuum of vanishing thickness adhered to the underlying bulk material without slipping [9]. The surface elasticity model has been successfully adopted to predict the size-dependent characteristics of many elastic nanostructures [10,11,12,13]. However, it fails to accurately describe the surface effects of piezoelectric nanostructures because the surface piezoelectricity is ignored. For this purpose, Huang and Yu [14] first proposed the surface piezoelectricity model, and Pan et al. [15] subsequently improved it. In this model, surface piezoelectricity and surface dielectricity are first introduced besides the surface elasticity and surface density involved in the surface elasticity model. Based on the surface piezoelectricity model, increased efforts have been conducted to obtain the static and dynamic responses of various piezoelectric nanostructures. To see concrete examples, one can refer to the comprehensive reviews carried out by Yan and Jiang [16] and Zhao et al. [17].

The design of SAW devices is mainly based on the propagation modes of Rayleigh or SH surface waves according to different working mechanisms [1,2,3]. Very recently, the authors examined the dispersion properties of Rayleigh-type surface waves propagating in a piezoelectric nanofilm attached to an elastic substrate, and we found that the surface effects have a considerable influence on the dispersion modes and phase velocity [18]. To enrich the studies on this topic, this work further investigates the propagation of SH surface waves in a similar layered piezoelectric nanostructure. Explicit expressions of the dispersion relationships are derived by using the surface piezoelectricity model, and the impacts of several surface-related parameters upon the dispersive behaviors are discussed in detail.

## 2. Problem Statement and Formulation

**Figure 1:**
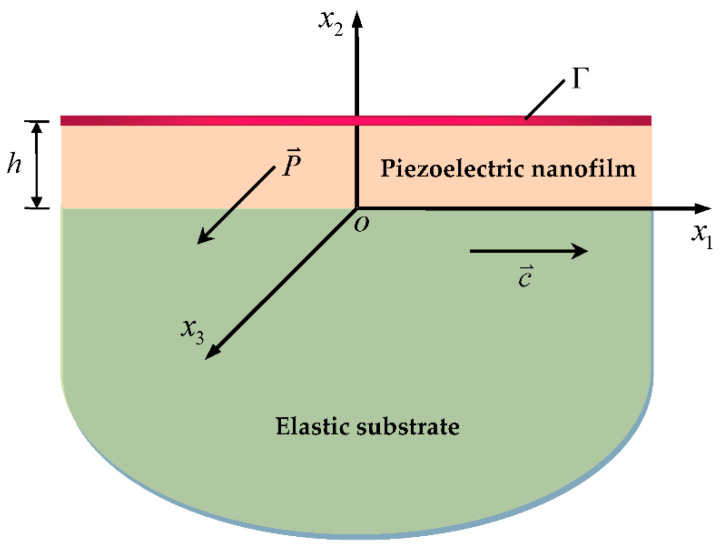
Geometry and coordinate systems of the problem.

The schematic of a layered piezoelectric nanostructure is depicted in Figure 1 along with the spatial rectangular coordinate system $ox_{1}x_{2}x_{3}$. A transversely isotropic piezoelectric nanofilm poling along the $x_{3}$-axis is perfectly bonded on an elastic substrate, which is modeled as an isotropic half-space in the theoretical analysis. The film thickness is h, and the surface at $x_{2}=h$ is denoted by plane Γ. When SH waves propagating in the $x_{1}x_{2}$-plane are considered, all field variables are independent of $x_{3}$, and only the out-of-plane mechanical displacement $u_{3}$ and the electric potential ϕ exist. Without the body forces and volume electric charges, the governing equations can be written as

(1) $$c_{44}^{E}\nabla^{2}u_{3}+e_{15}\nabla^{2}\phi =\rho {\ddot{u}}_{3},$$

(2) $$e_{15}\nabla^{2}u_{3}-\kappa_{11}^{\varepsilon }\nabla^{2}\phi =0,$$

for the piezoelectric film and

(3) $$\mu \nabla^{2}{\overset{⌣}{u}}_{3}=\overset{⌣}{\rho }{\ddot{\overset{⌣}{u}}}_{3},$$

(4) $$\nabla^{2}\overset{⌣}{\phi }=0,$$

for the elastic substrate. In Equations (1)–(4), $c_{44}^{E}$, $e_{15}$, $\kappa_{11}^{\varepsilon }$, and ρ are, respectively, the elastic stiffness measured at constant electric field, the piezoelectric constant, the dielectric permittivity measured at constant strain, and the mass density of the piezoelectric nanofilm; μ is the shear modulus of the elastic substrate, and $\nabla^{2}=\partial^{2}/\partial x_{1}^{2}+\partial^{2}/\partial x_{2}^{2}$ is the two-dimensional Laplacian operator. Throughout this paper, the overline is adopted to represent the substrate.

Using the separation of variables, the solutions to ${\overset{⌣}{u}}_{3}$ and $\overset{⌣}{\phi }$ satisfying Equations (3) and (4) take the form

(5) $${\overset{⌣}{u}}_{3}\left(x_{1},x_{2},t\right)=A_{1}\left[\cosh\left(k\overset{⌣}{\lambda }x_{2}\right)+\sinh\left(k\overset{⌣}{\lambda }x_{2}\right)\right]e^{ik\left(x_{1}-ct\right)},$$

(6) $$\overset{⌣}{\phi }\left(x_{1},x_{2},t\right)=A_{2}\left[\cosh\left(kx_{2}\right)+\sinh\left(kx_{2}\right)\right]e^{ik\left(x_{1}-ct\right)},$$

where $A_{i}$ is the unknown amplitudes, k is the wave number, c is the phase velocity, and $\overset{⌣}{\lambda }=\sqrt{1-{\left(c/{\overset{⌣}{c}}_{sh}\right)}^{2}}$, with ${\overset{⌣}{c}}_{sh}=\sqrt{\mu /\overset{⌣}{\rho }}$ being the bulk shear wave velocity of the elastic substrate. It is required that $\overset{⌣}{\lambda }>0$ in view of the attenuation condition $\underset{x_{2}\to -\infty }{\lim}({\overset{⌣}{u}}_{3},\overset{⌣}{\phi })=0$, so the phase velocity must satisfy

(7) $$c<{\overset{⌣}{c}}_{sh},$$

which implies that the substrate with a large velocity should be chosen in the high-frequency applications of SAW devices.

For the piezoelectric film, the out-of-plane displacement field is coupled with the in-plane electric field. Thus, $\psi =\phi -\gamma u_{3}$ with $\gamma =e_{15}/\kappa_{11}^{\varepsilon }$ is introduced to decouple the governing Equations (1) and (2) as

(8) $${\bar{c}}_{44}\nabla^{2}u_{3}=\rho {\ddot{u}}_{3},$$

(9) $$\nabla^{2}\psi =0,$$

in which ${\bar{c}}_{44}=c_{44}^{E}+e_{15}\gamma$ is the stiffened elastic constant due to the piezoelectric effect. The solutions of the above equations can be determined through the same procedure as that for the elastic substrate:

(10) $$u_{3}\left(x_{1},x_{2},t\right)=\left[A_{3}\cosh\left(k\lambda x_{2}\right)+A_{4}\sinh\left(k\lambda x_{2}\right)\right]e^{ik\left(x_{1}-ct\right)},\;\;\;\mathrm{when}\;c<c_{sh}<{\overset{⌣}{c}}_{sh},$$

(11) $$u_{3}\left(x_{1},x_{2},t\right)=\left[A_{3}\cos\left(k\lambda x_{2}\right)+A_{4}\sin\left(k\lambda x_{2}\right)\right]e^{ik\left(x_{1}-ct\right)},\;\;\;\mathrm{when}\;c_{sh}<c<{\overset{⌣}{c}}_{sh},$$

(12) $$\psi \left(x_{1},x_{2},t\right)=\left[A_{5}\cosh\left(kx_{2}\right)+A_{6}\sinh\left(kx_{2}\right)\right]e^{ik\left(x_{1}-ct\right)},$$

where $\lambda =\sqrt{\left|1-{\left(c/c_{sh}\right)}^{2}\right|}$, and $c_{sh}=\sqrt{{\bar{c}}_{44}/\rho }$ is the bulk shear wave velocity of the piezoelectric media. Then, the non-zero stress tensor σ and electric displacement vector D in the present case are obtained by

(13) $$\sigma_{23}={\bar{c}}_{44}u_{3,2}+e_{15}\psi_{,2},$$

(14) $$D_{2}=-\kappa_{11}^{\varepsilon }\psi_{,2},$$

(15) $${\overset{⌣}{\sigma }}_{32}=\mu {\overset{⌣}{u}}_{3,2},$$

(16) $${\overset{⌣}{D}}_{2}=-\eta {\overset{⌣}{\phi }}_{,2},$$

with η being the dielectric coefficient of the substrate.

Next, the surface effects are considered through utilizing the surface piezoelectricity model, which assumes that a surface possesses its own material properties and constitutive relationships different from those of its bulk counterpart. For the current formulation, the existing surface stress $\sigma_{31}^{s}$ and surface electric displacement $D_{1}^{s}$ obey

(17) $$\sigma_{31}^{s}=\sigma_{0}+{\bar{c}}_{44}^{s}u_{3,1}+e_{15}^{s}\psi_{,1},\;\;\;\;\;\;\;\;\;\;\mathrm{at}\;x_{2}=h,$$

(18) $$D_{1}^{s}=D_{0}+{\bar{e}}_{15}^{s}u_{3,1}-\kappa_{11}^{s}\psi_{,1},\;\;\;\;\;\;\;\;\;\;\mathrm{at}\;x_{2}=h,$$

with

(19) $${\bar{c}}_{44}^{s}=c_{44}^{s}+e_{15}^{s}\gamma ,\;\;\;\;\;\;\;\;\;\;{\bar{e}}_{15}^{s}=e_{15}^{s}-\kappa_{11}^{s}\gamma ,$$

where $\sigma_{0}$ and $D_{0}$ are the residual surface stress and surface electric displacement, and $c_{44}^{s}$, $e_{15}^{s}$, and $\kappa_{11}^{s}$ are the surface elastic, surface piezoelectric, and surface dielectric constants, respectively.

Unlike the classical situation, the presence of surface effects induces the jumps of stresses and electric displacements across the surface, which, under the anti-plane deformation, can be described by the nonclassical mechanical and electrical boundary conditions as [16,19,20]

(20) $$\sigma_{31,1}^{s}-\sigma_{32}=\rho_{s}{\ddot{u}}_{3},\;\;\;\;\;\;\;\;\;\;\mathrm{at}\;x_{2}=h,$$

(21) $$D_{1,1}^{s}-D_{2}=0,\;\;\;\;\;\;\;\;\;\;\mathrm{at}\;x_{2}=h,$$

where $\rho_{s}$ denotes the surface density. To be noted, these two equations may become the classical traction-free and electrically open-circuited case by vanishing all the surface-related quantities.

In principle, there exists interface effects at $x_{2}=0$, which also can be modeled by a method similar to the surface effects mentioned above. However, a class of unknown interface-related parameters needs to be introduced with the consideration of the interface effects, and it will unavoidably bring many difficulties to the computation and analysis. Accordingly, this paper focuses on examining the impacts of surface effects, while the interface effects are ignored. Consider that the mechanical and electric quantities are continuous along the interface, which requires

(22) $$u_{3}={\overset{⌣}{u}}_{3},\;\;\;\;\;\;\;\;\;\;\mathrm{at}\;x_{2}=0,$$

(23) $$\sigma_{32}={\overset{⌣}{\sigma }}_{32},\;\;\;\;\;\;\;\;\;\;\mathrm{at}\;x_{2}=0,$$

(24) $$\phi =\overset{⌣}{\phi },\;\;\;\;\;\;\;\;\;\;\;\;\mathrm{at}\;x_{2}=0,$$

(25) $$D_{2}={\overset{⌣}{D}}_{2},\;\;\;\;\;\;\;\;\;\;\mathrm{at}\;x_{2}=0.$$

Inserting the expressions of corresponding variables into Equations (20)–(25) results in the following system of homogeneous linear equations:

(26) $$Q_{11}A_{3}+Q_{12}A_{4}+Q_{13}A_{5}+Q_{14}A_{6}=0,$$

(27) $$Q_{21}A_{3}+Q_{22}A_{4}+Q_{23}A_{5}+Q_{24}A_{6}=0,$$

(28) $$A_{1}-A_{3}=0,$$

(29) $$\mu \overset{⌣}{\lambda }A_{1}-{\bar{c}}_{44}\lambda A_{4}-e_{15}A_{6}=0,$$

(30) $$A_{2}-\gamma A_{3}-A_{5}=0,$$

(31) $$\eta A_{2}-\kappa_{11}A_{6}=0,$$

where

(32) $$\begin{array}{c} Q_{11}=k\left({\bar{c}}_{44}^{s}-\rho_{s}c^{2}\right)\cosh\left(k\lambda h\right)+{\bar{c}}_{44}\lambda \sinh\left(k\lambda h\right),\\ Q_{12}=k\left({\bar{c}}_{44}^{s}-\rho_{s}c^{2}\right)\sinh\left(k\lambda h\right)+{\bar{c}}_{44}\lambda \cosh\left(k\lambda h\right),\\ Q_{21}={\bar{e}}_{15}^{s}k\cosh\left(k\lambda h\right),\\ Q_{22}={\bar{e}}_{15}^{s}k\sinh\left(k\lambda h\right), \end{array}\;\;\;\;\;\;\;\;\;\;\mathrm{when}\;c<c_{sh}<{\overset{⌣}{c}}_{sh},$$

(33) $$\begin{array}{c} Q_{11}=k\left({\bar{c}}_{44}^{s}-\rho_{s}c^{2}\right)\cos\left(k\lambda h\right)-{\bar{c}}_{44}\lambda \sin\left(k\lambda h\right),\\ Q_{12}=k\left({\bar{c}}_{44}^{s}-\rho_{s}c^{2}\right)\sin\left(k\lambda h\right)+{\bar{c}}_{44}\lambda \cos\left(k\lambda h\right),\\ Q_{21}={\bar{e}}_{15}^{s}k\cos\left(k\lambda h\right),\\ Q_{22}={\bar{e}}_{15}^{s}k\sin\left(k\lambda h\right), \end{array}\;\;\;\;\;\;\;\;\;\;\mathrm{when}\;c_{sh}<c<{\overset{⌣}{c}}_{sh},$$

and

(34) $$\begin{array}{c} Q_{13}=e_{15}^{s}k\cosh\left(kh\right)+e_{15}\sinh\left(kh\right),\\ Q_{14}=e_{15}^{s}k\sinh\left(kh\right)+e_{15}\cosh\left(kh\right),\\ Q_{23}=-\kappa_{11}^{s}k\cosh\left(kh\right)-\kappa_{11}^{\varepsilon }\sinh\left(kh\right),\\ Q_{24}=-\kappa_{11}^{s}k\sinh\left(kh\right)-\kappa_{11}^{\varepsilon }\cosh\left(kh\right). \end{array}$$

By eliminating $A_{1}$ and $A_{2}$ from Equations (28)–(31), one obtains

(35) $$A_{5}=\vartheta_{11}A_{3}+\vartheta_{12}A_{4},$$

(36) $$A_{6}=\vartheta_{21}A_{3}+\vartheta_{22}A_{4},$$

in which

(37) $$\begin{array}{l} \vartheta_{11}=\frac{\mu }{\gamma \eta }\overset{⌣}{\lambda }-\gamma ,\;\;\;\;\;\;\;\;\;\;\;\;\;\;\;\vartheta_{12}=-\frac{{\bar{c}}_{44}}{\gamma \eta }\lambda ,\\ \;\;\;\vartheta_{21}=\frac{\mu }{e_{15}}\overset{⌣}{\lambda },\;\;\;\;\;\;\;\;\;\;\;\;\;\;\;\;\;\vartheta_{22}=-\frac{{\bar{c}}_{44}}{e_{15}}\lambda . \end{array}$$

Then, substitution of Equations (35) and (36) into Equations (26) and (27) yields

(38) $$\Delta_{1}A_{3}+\Delta_{2}A_{4}=0,$$

(39) $$\Delta_{3}A_{3}+\Delta_{4}A_{4}=0,$$

with

(40) $$\begin{array}{l} \Delta_{1}=Q_{11}+Q_{13}\vartheta_{11}+Q_{14}\vartheta_{21},\\ \Delta_{2}=Q_{12}+Q_{13}\vartheta_{12}+Q_{14}\vartheta_{22},\\ \Delta_{3}=Q_{21}+Q_{23}\vartheta_{11}+Q_{24}\vartheta_{21},\\ \Delta_{4}=Q_{22}+Q_{23}\vartheta_{12}+Q_{24}\vartheta_{22}. \end{array}$$

To obtain the nontrivial solutions of Equations (38) and (39), the determinant of coefficient matrix must vanish, i.e.,

(41) $$\left|\begin{array}{cc} \Delta_{1} & \Delta_{2}\\ \Delta_{3} & \Delta_{4} \end{array}\right|=\Delta_{1}\Delta_{4}-\Delta_{2}\Delta_{3}=0.$$

The dispersion relationships between the phase velocity c and the wave number k can be further determined by solving Equation (41) numerically. It is worth noting that both the residual surface stress $\sigma_{0}$ and surface electric displacement $D_{0}$ are absent in Equation (41), which means that these two residual fields have no effect on the dispersion behaviors of SH-type surface waves. This is consistent with the theoretical results given earlier by Gurtin and Murdoch [21]. Specifically, when the surface effects are excluded by setting all the surface-related parameters equal to zero, Equations (20) and (21) are reduced to the conventional traction-free and electrically open-circuited boundary conditions, under which Equation (41) takes the following form since the solutions exist only if $c_{sh}<c<{\overset{⌣}{c}}_{sh}$, i.e.,

(42) $${\bar{c}}_{44}\lambda \tan\left(\lambda kh\right)=\mu \overset{⌣}{\lambda }+\frac{\gamma^{2}\eta \tanh\left(kh\right)}{\tanh\left(kh\right)+\eta /\kappa_{11}^{\varepsilon }},$$

which corresponds to classical Love waves and is identical to that obtained by Li et al. [22].

Consider the two limit cases of wave number below. First, setting k→0, both of Equations (41) and (42) are simplified to

(43) $$c={\overset{⌣}{c}}_{sh},\;\;\;\;\;\;\;\;\;\;\mathrm{when}\;c_{sh}<c<{\overset{⌣}{c}}_{sh},$$

and the surface effects in Equation (41) are invalid at this point. This is because the waves mainly propagate in the semi-infinite substrate at very low frequencies, and apparently, the surface effects of piezoelectric film have no impact on the wave characteristics of the substrate. Next, taking the limit k→∞ in Equation (41) under the case of $c<c_{sh}<{\overset{⌣}{c}}_{sh}$ results in

(44) $$\lambda =\frac{k\left(\rho_{s}c^{2}-{\bar{c}}_{44}^{s}\right)\left(\kappa_{11}^{s}k+\kappa_{11}^{\varepsilon }\right)-{\bar{e}}_{15}^{s}k\left(e_{15}^{s}k+e_{15}\right)}{{\bar{c}}_{44}\left(\kappa_{11}^{s}k+\kappa_{11}^{\varepsilon }\right)}.$$

## 3. Numerical Results and Discussion

As a case study, consider a piezoelectric nanofilm of PZT-5H on an elastic substrate of diamond, whose material constants used for the numerical calculation are listed in Table 1 [23,24]. For the piezoelectric surface, since the material properties that can be determined from atomistic simulations or experiments are not completely available in the literatures owing to lack of such work, a simple but reasonable method is proposed to define each surface material constant as a scaled version of its bulk counterpart. Therefore, the following relationships are introduced, i.e.,

(45) $$c_{44}^{s}=f_{c}c_{44}^{E},\;\;\;\;\;e_{15}^{s}=f_{e}e_{15},\;\;\;\;\;\kappa_{11}^{s}=f_{\kappa }\kappa_{11}^{\varepsilon },\;\;\;\;\;\rho_{s}=f_{\rho }\rho ,$$

where $f_{c}$, $f_{e}$, $f_{\kappa }$, and $f_{\rho }$ are the nanoscaled characteristic lengths representing the magnitudes of surface elasticity, surface piezoelectricity, surfacedielectricity, and the surface density, respectively. The dimensionless frequency $K=kch/c_{sh}$ and phase velocity $c/c_{sh}$ are adopted for simplicity, and both of them are assumed to be real and positive. That is to say, this paper only involves the time-harmonic traveling waves that are not attenuating. To be noted, we take h=2 nm in the following analysis unless otherwise stated.

**Figure 2:**
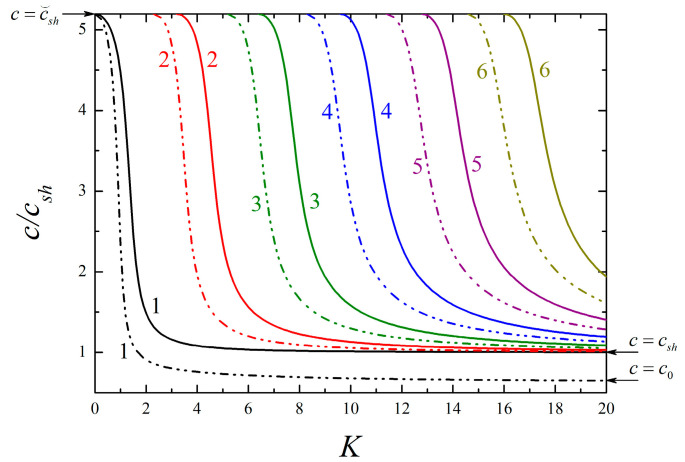
Dispersion curves of the lowest six modes for SH-type surface waves in a piezoelectric film/elastic substrate structure. Dashed–dotted lines indicate the case of the nanostructure with *f_c_* = *f_e_* = 0.5 nm and *f_κ_* = *f_ρ_* = 1 nm, and solid lines indicate the case of the corresponding classical structure with *f_c_* = *f_e_* = *f_κ_* = *f_ρ_* = 0 nm.

**Figure 3:**
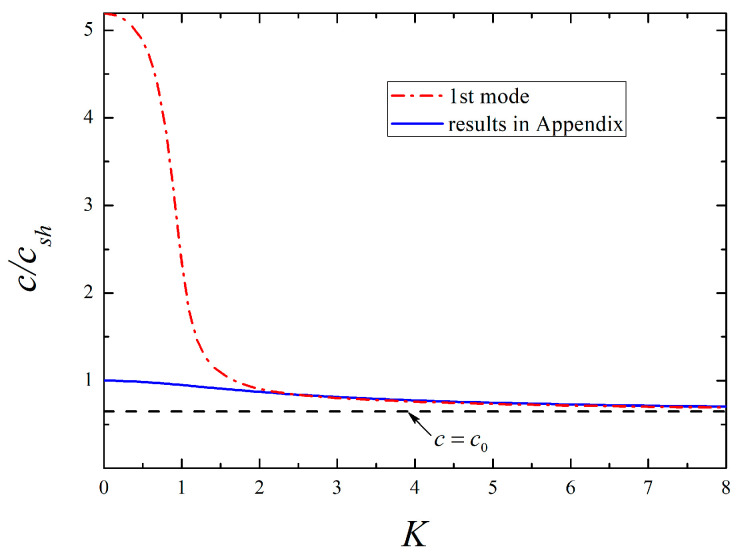
Comparison of the 1st mode for the present nanostructure and the SH surface waves for a piezoelectric half-space with surface effects.

Following Equations (41) and (42), respectively, the dispersion curves of the lowest six modes for SH-type surface waves are plotted in Figure 2, where the 1st mode, known as fundamental mode, exists under arbitrary low frequency, while the high-order ones propagate only above the individual cut-off frequency corresponding to c→∞. It is evident that the presence of surface effects results in a marked departure from the classical case, and this implies the necessity of incorporating surface effects into the wave analysis of piezoelectric nanostructures. Whether the surface effects are included or not, the phase velocities of all modes descend rapidly from ${\overset{⌣}{c}}_{sh}$, with the frequency K increasing. Then, they all approach $c_{sh}$ as K grows high enough except in the 1st mode considering the surface effects. Since the 1st mode propagates as in a piezoelectric half-space when K→∞, the propagation of SH waves in such a structure is discussed in the Appendix A. In contrast to the classical case that no SH-type surface waves exist, one observes that such waves can occur under a particular condition (see Equation (A13)) due to the existence of surface effects, and they are dispersive with a lower velocity than $c_{sh}$. The phase velocity curve based on Equation (A8) is also depicted in Figure 3 to compare with the 1st mode. It is seen that two curves converge at higher frequencies, and the velocities are asymptotic to a certain value $c_{0}$ that is completely determined by the surface-related material parameters as K→∞. This phenomenon indicates the 1st mode may propagate in the form of SH surface waves at a high-frequency range, which is also confirmed in theory because the expression of Equation (A8) is identical to Equation (44). In addition, Figure 2 shows that the surface effects have a greater influence on the high-order modes with the increase of modes order.

Within the surface piezoelectricity model, surface effects actually synthesize the impacts of surface elasticity, surface piezoelectricity, surface dielectricity, and the surface density. Each of these impacts on the dispersive modes will be provided individually with selected surface parameters in what follows. Without loss of generality, the 2nd mode is included to represent the high-order modes.

**Figure 4:**
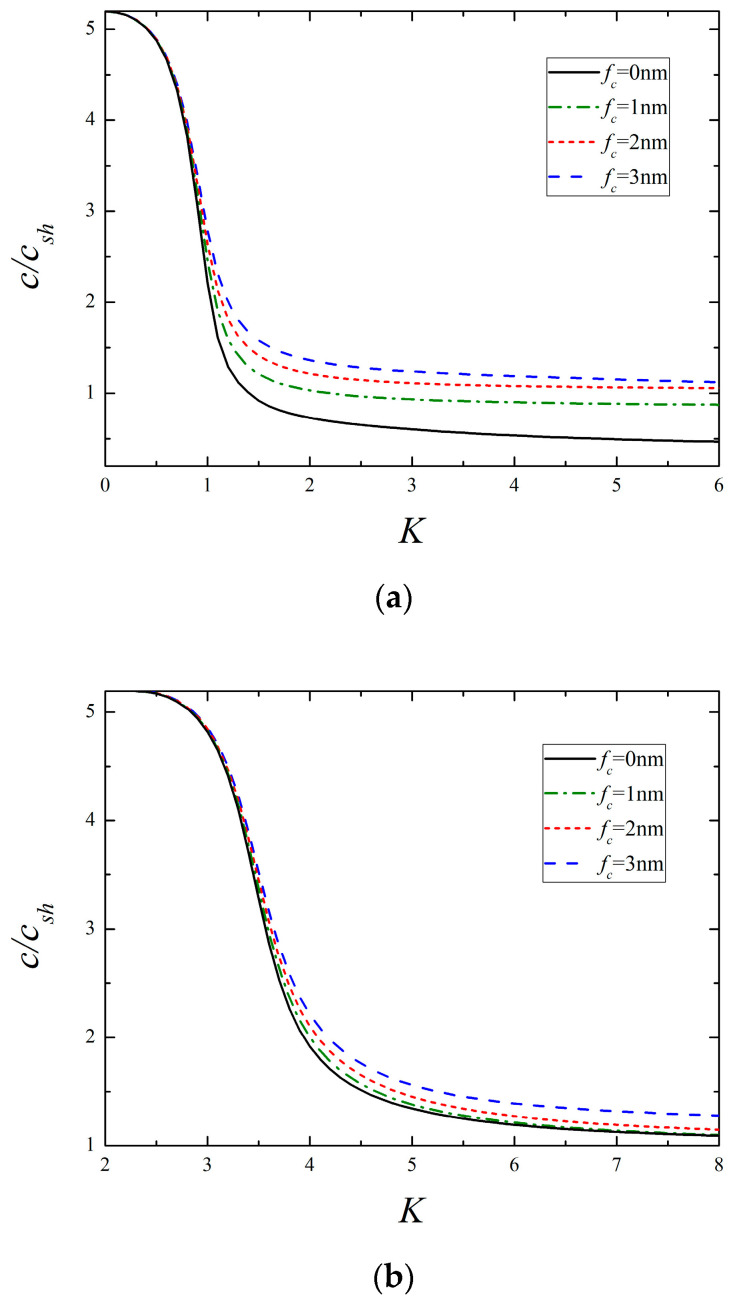
The lowest two modes for different surface elasticity (*f_e_* = 0.5 nm, *f_κ_* = *f_ρ_* = 1 nm). (**a**) The 1st mode and (**b**) the 2nd mode.

**Figure 5:**
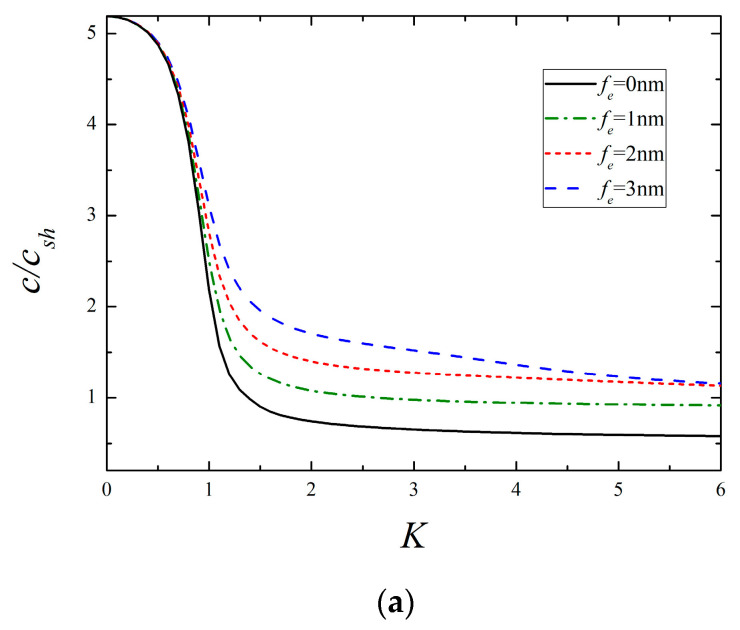

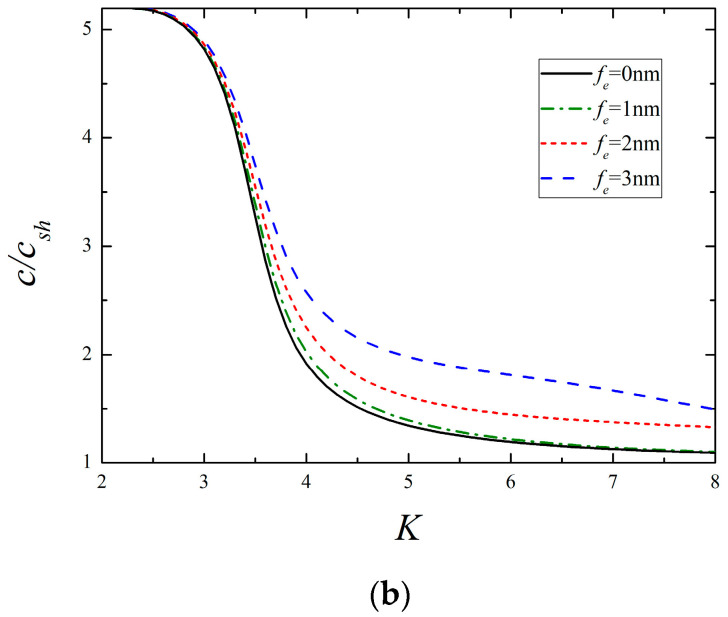
The lowest two modes for different surface piezoelectricity (*f_c_* = 0.5 nm, *f_κ_* = *f_ρ_* = 1 nm). (**a**) The 1st mode and (**b**) the 2nd mode.

Figure 4 and Figure 5 depict the dispersion curves of the lowest two modes for different surface elasticity and surface piezoelectricity, respectively. Regardless of the 1st or 2nd mode, it is observed that the presence of surface elasticity causes the increase of phase velocity, and the larger the value of $f_{c}$, the higher the phase velocity becomes. A close comparison of Figure 4 and Figure 5 reveals that the influencing mechanism of surface piezoelectricity upon the corresponding mode is like that of surface elasticity. If $f_{c},\;f_{e}=2$ or 3 nm, it should be pointed out that the condition (A13) in Appendix A is unsatisfied, then the phase velocity of the 1st mode tends to $c_{sh}$ as K→∞, which is the same as the classical situation shown in Figure 2. Similar events also occur in the following examples. Together with the aforementioned results, it concludes that the 1st mode at high frequencies is SH bulk waves or surface waves strongly depending on the relative value of surface material parameters.

**Figure 6:**
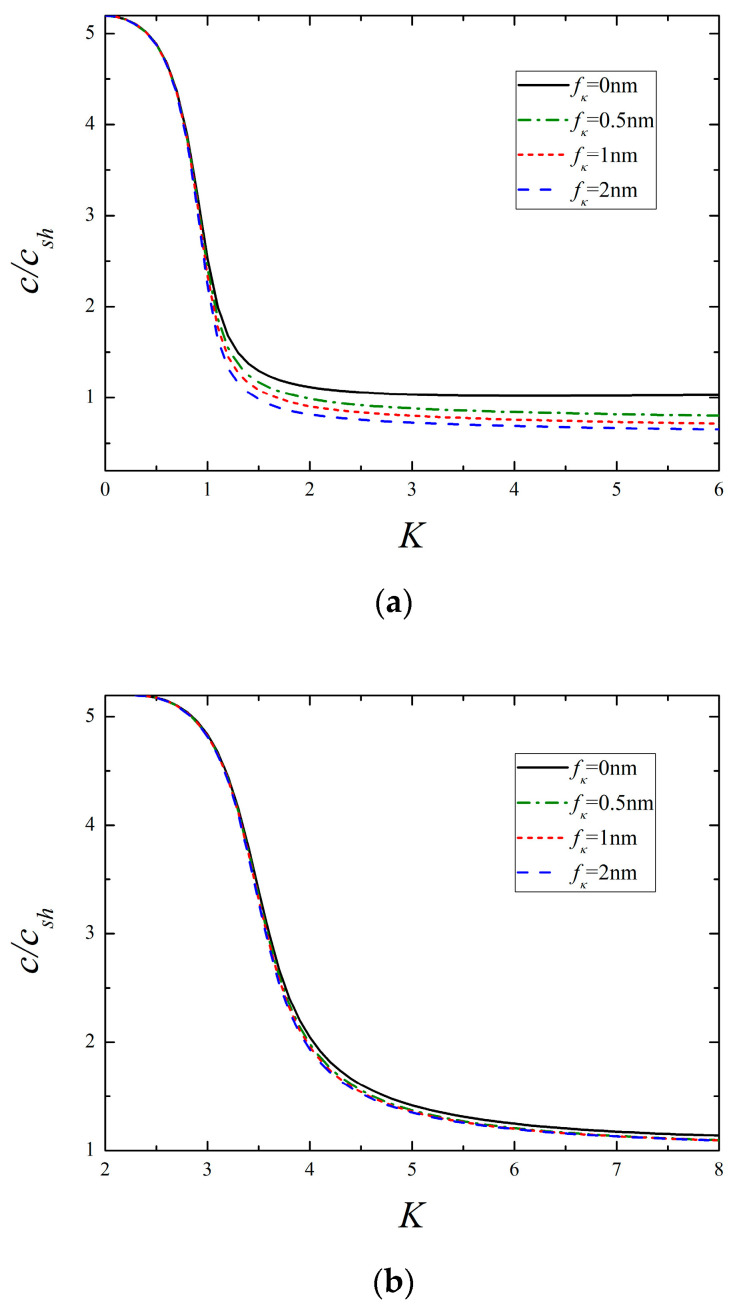
The lowest two modes for different surface dielectricity (*f_c_* = *f_e_* = 0.5 nm, *f_ρ_* = 1 nm). (**a**) The 1st mode and (**b**) the 2nd mode.

**Figure 7:**
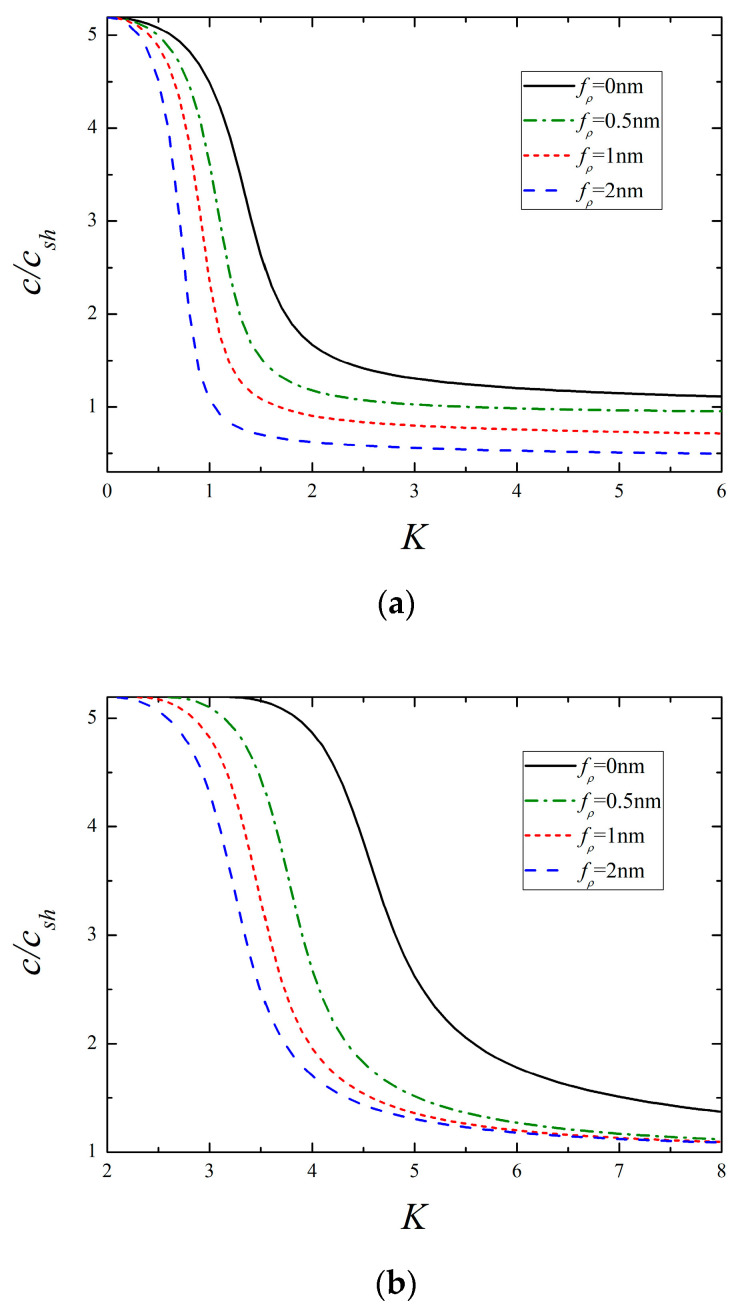
The lowest two modes for different surface density (*f_c_* = *f_e_* = 0.5 nm, *f_κ_* = 1 nm). (**a**) The 1st mode and (**b**) the 2nd mode.

Dispersion curves of the lowest two modes for different surface dielectricity are illustrated in Figure 6. One can see that the phase velocity declines with the growing value of $f_{\kappa }$, and the variation is remarkable for the 1st mode, whereas it is slight for the 2nd mode. By inspection of Figure 4, Figure 5 and Figure 6, it is found that these three surface parameters making a difference in the phase velocity is more significant at higher frequencies than that at lower frequencies. Similar plots for different surface densities are presented in Figure 7. It is clear that a larger value of $f_{\rho }$ produces a lower wave velocity, especially in the low-frequency range, which is different from the influence of the former three parameters. Another important finding from Figure 7b is that the cut-off frequency of the 2nd mode is greatly lowered in the presence of surface density, while it remains constant as the other surface parameters vary by itself, as shown in Figure 4b, Figure 5b and Figure 6b. It indicates that the change of cut-off frequency attributed to the surface effects is completely dominated by the surface density. The reduction of cut-off frequency extends the frequency domain of high-order modes, which may provide useful guidance for selecting the frequency and mode. For instance, if only the 1st mode is expected in practical application, the smaller exciting frequency should be used in view of the above conclusion.

**Figure 8:**
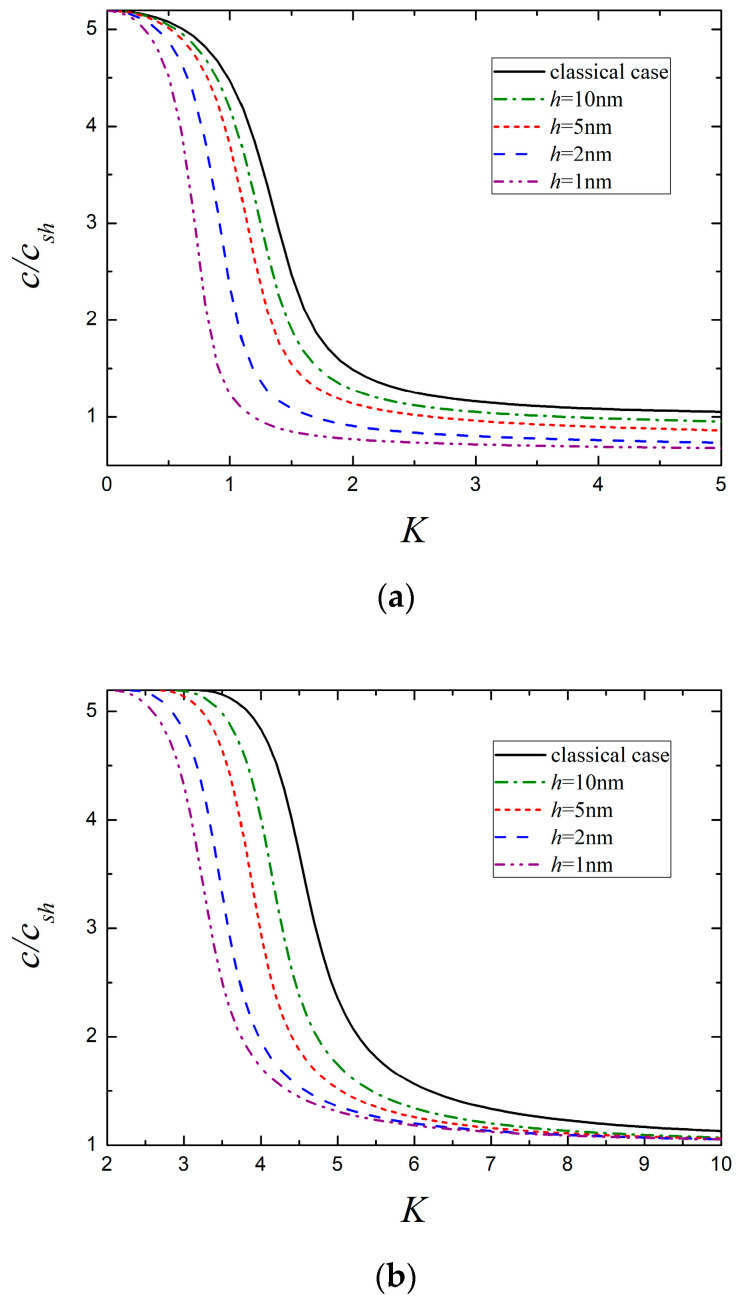
The lowest two modes for different nanofilm thickness. (**a**) The 1st mode and (**b**) the 2nd mode.

The variation of the dispersion curves of the lowest two modes with different film thickness is plotted in Figure 8, where the classical results for which curve remains unchanged are also plotted for comparison. It is obvious that the presence of surface effects gives rise to size-dependent dispersive behaviors, and a smaller film thickness corresponds a larger deviation from the classical case. This coincides with the fact that the surface effects become more significant as the size of nanostructure decreases. In addition, it can be reasonably deduced from Figure 8 that the surface effects may disappear once the thickness of piezoelectric nanofilm reaches a critical value.

It should be mentioned that in addition to the surface piezoelectricity theory, in recent years, several higher-order continuum mechanics theories such as nonlocal theory [25,26], coupled stress theory [27], flexoelectric theory [28], and strain gradient theory [29] also have been developed to successfully capture the size-dependent wave properties of piezoelectric nanostructures from different perspectives.

## 4. Conclusions

Based on the surface piezoelectricity model, this paper investigates the dispersion properties of SH-type surface waves propagating in a layered piezoelectric nanostructure composed of a piezoelectric nanofilm and an elastic half-space. Numerical analysis is performed to show the impacts of several surface-related material parameters on the dispersion modes and wave velocity. The main findings are summarized as follows:

(1) As opposed to the surface dielectricity and surface density, the enhanced surface elasticity and surface piezoelectricity increase the phase velocity. The expression of $c_{0}$ in Equation (A11) may provide some theoretic supports for this conclusion, in which the former two parameters are located on the denominator and the latter on the numerator.
(2) The effects of all surface parameters are considerable at higher frequencies, while only the surface density plays a prominent role at lower frequencies.
(3) The cut-off frequencies of high-order modes are dependent on the surface density rather than the other surface parameters.
(4) The 1st mode propagating at higher frequencies in the form of bulk waves or surface waves is dominated by the relative value of surface material parameters.
(5) Size-dependent dispersion properties occurring with the surface effects are predicted, and they may vanish when the film thickness exceeds a critical value.

These results could help increase the understanding of the wave characteristics of piezoelectric nanostructures and provide guidelines for the design and applications of smart nanodevices.

## Figures and Tables

**Table 1:** Material constants of PZT-5H and diamond used for the numerical calculation [23,24].

Material Constants	Symbol	PZT-5H	Diamond
Elastic constants (10^9^ N/m^2^)	$c_{44}^{E}$	23	—
	μ	—	533.3
Piezoelectric constants (C/m^2^)	$e_{15}$	17	—
Dielectric constants (10^−11^ C/Vm)	$\kappa_{11}^{\varepsilon }$	1505	—
	η	—	5.02
Density (kg/m^3^)	ρ	7500	3512

## Appendix A. Existence of SH Surface Waves in a Piezoelectric Half-Space with Considering Surface Effects

For a piezoelectric body occupying half-space $x_{2}\geq 0$, when SH waves propagation along the $x_{1}$ direction is considered, the general solutions to Equations (1) and (2) are given by

(A1) $$u_{3}\left(x_{1},x_{2},t\right)=B_{1}e^{-k\hat{\lambda }x_{2}}e^{ik\left(x_{1}-ct\right)},$$

(A2) $$\psi \left(x_{1},x_{2},t\right)=B_{2}e^{-kx_{2}}e^{ik\left(x_{1}-ct\right)},$$

where $B_{i}\left(i=1,2\right)$ is unknown amplitude, and $\hat{\lambda }=\sqrt{1-{\left(c/c_{sh}\right)}^{2}}$ must remain positive in view of the attenuation condition $\underset{x_{2}\to +\infty }{\lim}u_{3}=0$, which requires

(A3) $$c<c_{sh}.$$

With the consideration of surface effects, the boundary conditions along the surface are expressed as

(A4) $$\sigma_{31,1}^{s}+\sigma_{32}=\rho_{s}{\ddot{u}}_{3},\;\;\;\;\;\;\;\;\;\;\mathrm{at}\;x_{2}=0,$$

(A5) $$D_{1,1}^{s}+D_{2}=0,\;\;\;\;\;\;\;\;\;\;\mathrm{at}\;x_{2}=0,$$

Substituting Equations (A1) and (A2) into (A4) and (A5) with the help of Equations (13) and (14) as well as (17) and (18), one obtains

(A6) $$A\left[k\left({\bar{c}}_{44}^{s}-\rho_{s}c^{2}\right)+{\bar{c}}_{44}\lambda \right]+B\left(e_{15}^{s}k+e_{15}\right)=0,$$

(A7) $$A{\bar{e}}_{15}^{s}k-B\left(\kappa_{11}^{s}k+\kappa_{11}^{\varepsilon }\right)=0,$$

The requirement that the determinant of the coefficient matrix of Equations (A6) and (A7) vanish leads to

(A8) $$\lambda =\frac{k\left(\rho_{s}c^{2}-{\bar{c}}_{44}^{s}\right)\left(\kappa_{11}^{s}k+\kappa_{11}^{\varepsilon }\right)-{\bar{e}}_{15}^{s}k\left(e_{15}^{s}k+e_{15}\right)}{{\bar{c}}_{44}\left(\kappa_{11}^{s}k+\kappa_{11}^{\varepsilon }\right)},$$

whose expression is exactly the same to Equation (44). Clearly, Equation (A8) shows the dependence of phase velocity on wave number; thus, these SH waves are dispersive. When the surface effects are absent, Equation (A8) reduces to λ=0 that requires $c=c_{sh}$, which proves no SH surface waves exist in the classical case.

In view of λ>0, one obtains from Equation (A8)

(A9) $$c>\sqrt{\frac{{\bar{c}}_{44}^{s}+g\left(k\right)}{\rho_{s}}},$$

where $g\left(k\right)=\frac{{\bar{e}}_{15}^{s}\left(e_{15}^{s}k+e_{15}\right)}{\kappa_{11}^{s}k+\kappa_{11}^{\varepsilon }}$ is the monotonically increasing function of k because dg/dk≥0, and its maximum is

(A10) $$\max\left[g\left(k\right)\right]=\underset{k\to \infty }{\lim}g\left(k\right)=\frac{{\bar{e}}_{15}^{s}e_{15}^{s}}{\kappa_{11}^{s}}.$$

We further let

(A11) $$c_{0}=\sqrt{\frac{{\bar{c}}_{44}^{s}+\max\left[g\left(k\right)\right]}{\rho_{s}}}=\sqrt{\frac{{\bar{c}}_{44}^{s}+\frac{{\bar{e}}_{15}^{s}e_{15}^{s}}{\kappa_{11}^{s}}}{\rho_{s}}},$$

with $c_{0}$ being the asymptotic solution of Equation (A8) to the phase velocity when k→∞. Then, together with Equations (A3), (A9), and (A11), this yields

(A12) $$c_{sh}>c>c_{0},$$

which means the SH waves possibly propagate along the surface only if

(A13) $$c_{sh}>c_{0}.$$

Since $c=c_{sh}$ corresponds to the limit k→0, the dispersion curve governed by Equation (A8) must start from $c_{sh}$ to $c_{0}$ as the wave number increases, as shown in Figure 3.

When the piezoelectric effect is neglected, i.e., setting $e_{15}=e_{15}^{s}=0$ in Equation (A13), the existence condition of SH surface waves in an isotropic half-space with surface effects is obtained as

(A14) $$c_{sh}^{e}>c_{0}^{e},$$

which is identical to that obtained by Murdoch [30] with

(A15) $$c_{sh}^{e}=\sqrt{\frac{c_{44}^{E}}{\rho }},\;\;\;\;\;\;\;\;\;\;c_{0}^{e}=\sqrt{\frac{c_{44}^{s}}{\rho_{s}}},$$

where $c_{sh}^{e}$ is the bulk shear wave velocity of the isotropic media.

It should be mentioned that the condition (A13) is invalid if all the surface material parameters are homogenized, i.e., $f_{c}=f_{e}=f_{\kappa }=f_{\rho }\neq 0\;\mathrm{nm}$. For the isotropic case mentioned above, Murdoch pointed out that a departure from homogeneity is to be expected in the surface region for practical situations [30]. From this, the homogenized surface parameters are excluded in the numerical analysis.

## References

[1] Fu Y., Luo J., Du X., Flewitt A., Li Y., Markx G., Walton A., Milne W. (2010). Recent developments on ZnO films for acoustic wave based bio-sensing and microflfluidic applications: A review. Sensor. Actuat. B Chem..

[2] Hadj-Larbi F., Serhane R. (2019). Sezawa SAW devices: Review of numerical-experimental studies and recent applications. Sensor. Actuat. A Phys..

[3] Huang Y., Das P., Bhethanabotla V. (2021). Surface acoustic waves in biosensing applications. Sensor. Actuat. Rep..

[4] Wu S., Ro R., Lin Z., Lee M. (2009). High velocity shear horizontal surface acoustic wave modes of interdigital transducer/(100) AlN/(111) diamond. Appl. Phys. Lett..

[5] Wu S., Ro R., Lin Z., Lee M. (2008). Rayleigh surface acoustic wave modes of interdigital transducer/(100)AIN/(111) diamond. J. Appl. Phys..

[6] Liu J., Wang Y., Wang B. (2010). Propagation of shear horizontal surface waves in a layered piezoelectric half-space with an imperfect interface. IEEE Trans. Ultrason. Ferroelectr. Freq. Control.

[7] Yamanaka K., Ishikawa S., Nakaso N., Takeda N., Sim D., Mihara T., Mizukami A., Satoh I., Akao S., Tsukahara Y. (2006). Ultramultiple roundtrips of surface acoustic wave on sphere realizing innovation of gas sensors. IEEE Trans. Ultrason. Ferroelectr. Freq. Control.

[8] Nakatsukasa T., Akao S., Ohgi T., Nakaso N., Abe T., Yamanaka K. (2006). Temperature compensation for ball surface acoustic wave devices and sensor using frequency dispersion. Jpn. J. Appl. Phys..

[9] Gurtin M., Murdoch A. (1975). A continuum theory of elastic material surfaces. Arch. Ration. Mech. Anal..

[10] Wang J., Huang Z., Duan H., Yu S., Feng X., Wang G., Zhang W., Wang T. (2011). Surface stress effect in mechanics of nanostructured materials. Acta Mech. Solida Sin..

[11] Assadi A., Farshi B., Alinia-Ziazi A. (2010). Size dependent dynamic analysis of nanoplates. J. Appl. Phys..

[12] Chakraborty A. (2010). The effect of surface stress on the propagation of Lamb waves. Ultrasonics.

[13] Zhu F., Pan E., Qian Z., Wang Y. (2019). Dispersion curves, mode shapes, stresses and energies of SH and Lamb waves in layered elastic nanoplates with surface/interface effect. Int. J. Eng. Sci..

[14] Huang G., Yu S. (2006). Effect of surface piezoelectricity on the electromechanical behavior of a piezoelectric ring. Phys. Status Solidi B.

[15] Pan X., Yu S., Feng X. (2011). A continuum theory of surface piezoelectricity for nanodielectrics. Sci. China Ser. G Phys. Mech. Astron..

[16] Yan Z., Jiang L. (2017). Modified continuum mechanics modeling on size-dependent properties of piezoelectric nanomaterials: A review. Nanomaterials.

[17] Zhao Z., Zhu J., Chen W. (2022). Size-dependent vibrations and waves in piezoelectric nanostructures: A literature review. Int. J. Smart Nano Mat..

[18] Zhang L., Zhao J., Nie G., Liu J. (2022). Propagation of Rayleigh-type surface waves in a layered piezoelectric nanostructure with surface effects. Appl. Math. Mech-Engl..

[19] Chen T., Chiu M., Weng C. (2006). Derivation of the generalized Young-Laplace equation of curved interfaces in nanoscaled solids. J. Appl. Phys..

[20] Zhang L., Liu J., Nie G., Fang X. (2014). Effects of surface piezoelectricity and nonlocal scale on wave propagation in piezoelectric nanoplates. Eur. J. Mech. A-Solid..

[21] Gurtin M., Murdoch A. (1976). Effect of surface stress on wave propagation in solids. J. Appl. Phys..

[22] Li P., Jin F., Lu T. (2012). A three-layer structure model for the effect of a soft middle layer on Love waves propagating in layered piezoelectric systems. Acta Mech. Sinica.

[23] Yang J. (2006). Analysis of Piezoelectric Devices.

[24] Benetti M., Cannata D., Di-Pietrantonio F., Verona E. (2005). Growth of ALN piezoelectric film on diamond for high-frequency surface acoustic wave devices. IEEE Trans. Ultrason. Ferroelectr. Freq. Control.

[25] Wang X., Ren X., Yu J., Zhang X., Zhang B. (2022). Escape, crossing and cut-off frequencies of SH waves in nonlocal piezoelectric nanoplates. Thin Wall. Struct..

[26] Liu C., Yu J., Zhang B., Wang X., Zhang X., Zhang H. (2022). Complete guided wave in piezoelectric nanoplates: A nonlocal stress expansion polynomial method. Eur. J. Mech. A-Solid..

[27] Liu C., Yu J., Zhang X., Zhang B., Elmaimouni L. (2020). Reflection behavior of elastic waves in the functionally graded piezoelectric microstructures. Eur. J. Mech. A-Solid..

[28] Yang W., Liang X., Shen S. (2017). Love waves in layered flexoelectric structures. Philos. Mag..

[29] Arefi M., Zenkour A. (2016). Free vibration, wave propagation and tension analyses of a sandwich micro/nano rod subjected to electric potential using strain gradient theory. Mater. Res. Express.

[30] Murdoch A. (1976). The propagation of surface waves in bodies with material boundaries. J. Mech. Phys. Solids.

